# Diet Quality as an Associated Factor for Liver Function Enzyme Abnormalities Among Women of Reproductive Age

**DOI:** 10.7759/cureus.102608

**Published:** 2026-01-29

**Authors:** Fatehatun Noor, Nusrat Jahan Shorovi, Md. Mahadi Hasan Shoruve, Tasmim Fahima Ahmad, FNU Asamoni, Nisarga Bahar, Md. Ruhul Amin, Tanjina Rahman, Abu Ahmed Shamim, M Akhtaruzzaman

**Affiliations:** 1 Institute of Nutrition and Food Science, University of Dhaka, Dhaka, BGD; 2 Department of Food Science and Nutrition, Hajee Mohammad Danesh Science and Technology University, Dinajpur, BGD; 3 Department of Life Science, Independent University, Bangladesh, Dhaka, BGD; 4 Department of Epidemiology, Bangladesh University of Health Sciences, Dhaka, BGD; 5 BRAC James P Grant School of Public Health, BRAC University, Dhaka, BGD

**Keywords:** alanine aminotransferase (alt), aspartate aminotransferase (ast), dietary diversity (mdd-w), diet quality, food consumption score (fcs), women of reproductive age

## Abstract

Background

Elevated liver enzymes, such as alanine aminotransferase (ALT) and aspartate aminotransferase (AST), are early indicators of liver dysfunction in non-alcoholic fatty liver disease (NAFLD). The prevalence of NAFLD is increasing in Bangladesh, with studies indicating a higher prevalence among women. Additionally, rapid dietary transitions toward energy-dense foods rich in fat and sugar have contributed to the rising burden of non-communicable diseases. This study aimed to investigate the association between dietary intake, diet quality indicators, and elevated liver enzymes (ALT and AST) among women of reproductive age in Bangladesh.

Methods

A cross-sectional study was conducted among 240 women of reproductive age (15-49 years) randomly selected from community households in three selected districts of Bangladesh. Anthropometric and socioeconomic data were collected using standardized tools. Dietary intake was assessed using a single 24-hour dietary recall method and a Food Frequency Questionnaire (FFQ). Three complementary indices were applied to evaluate diet quality: Dietary Diversity (DD), Food Consumption Score (FCS), and the Bangladesh Healthy Eating Index (BD_HEI). The participant's blood was collected after overnight fasting. Serum ALT and AST levels were measured using a biochemical analyzer, and elevations in liver enzymes were defined according to standard clinical cut-off values. Descriptive and inferential statistics were performed using STATA version 15.1 (Stata Corp LLC, College Station, TX, USA).

Results

Among the 240 participants, 69 (28.7%) exhibited elevated ALT and/or AST levels, while 171 (71.3%) had normal values. Participants with elevated liver enzymes had higher intake of saturated fat (5.89 vs. 4.41 g, IQR: (3.84-9.63 vs. 2.98-7.15), *p* =0.010), cholesterol (55.21 vs. 30.41 mg, IQR: (15.21-137.92 vs. 0-106.61), *p* = 0.015). Participants with elevated liver enzymes had significantly lower FCS scores (23.2% vs. 9.4%, *p* = 0.014) but higher DD (27.5% vs. 15.8%, *p* = 0.036) as compared to normal liver enzymes. Multivariable logistic regression revealed that low FCS significantly increased the odds of elevated liver enzymes (AOR = 3.64, 95% CI: 1.53-8.62, *p* = 0.003).

Conclusion

This study highlights a significant association between poor FCS and liver enzyme abnormalities among Bangladeshi women of reproductive age. Integrating dietary quality interventions into reproductive health and noncommunicable disease (NCD) prevention programs could play a vital role in maintaining liver health and metabolic well-being in this vulnerable population.

## Introduction

The liver plays a vital role in digestion, protein synthesis, detoxification, lipid metabolism, and glucose homeostasis [1]. Previous studies in Bangladesh have reported non-alcoholic fatty liver disease (NAFLD) prevalence ranging from 18.5% in a community-based survey to 33.8% among adult volunteers, with both studies consistently showing a higher prevalence among females than males [2,3]. Elevated liver enzymes such as alanine aminotransferase (ALT) and aspartate aminotransferase (AST) are important biomarkers of NAFLD. Previous studies have reported elevated ALT prevalence of 18.8% and AST of 21.6% among general adults [4], with one study involving only female participants found that the elevated ALT and AST were 21.5% and 18.7%, respectively [5].

Dietary patterns and nutrient intake are strongly linked to liver health [6]. High prevalence of vitamin B12, zinc [7], and tocopherol [8] deficiencies has also been reported among pregnant women from Bangladesh. Micronutrient deficiencies, as well as excessive intakes of saturated fats, trans fats, refined sugars, and animal proteins, can contribute to abnormal liver function through mechanisms involving triglyceride accumulation, oxidative stress, and insulin resistance [9]. Similar associations have been observed in Indian and Bangladeshi populations, where dietary intake is a major predictor of liver-related non-communicable diseases [10]. Dietary studies in various populations have shown that low intakes of fruits, vegetables, and milk, combined with higher intakes of red meat, fats, and energy-dense foods, are associated with elevated liver enzymes [11].

Diet quality indices such as Dietary Diversity (MDD-W), Food Consumption Score (FCS), and Bangladesh Healthy Eating Index (BD_HEI) have been used globally to assess nutrient adequacy and diet-disease relationships. DD reflects micronutrient adequacy [12], while FCS evaluates household food access and consumption [13]. However, the relationship between DD and non-communicable diseases is complex and depends on the types of foods consumed. In some settings, increased dietary diversity has been associated with higher fat intake, which may contribute to metabolic disorders [14]. A low FCS may be associated with an increased risk of developing noncommunicable diseases (NCDs), including abnormalities such as elevated liver enzyme levels [15]. HEI assesses adherence to dietary guidelines and has been linked to lower rates of elevated liver enzymes [16] through higher fruit and vegetable consumption [17].

Despite these global insights, there is limited research exploring how dietary intake, FCS, is associated with liver enzyme abnormalities among Bangladeshi women of reproductive age. This study aims to address this gap by examining the relationship between diet quality indicators and elevated liver function markers in this population.

## Materials and methods

Study design and population

This cross-sectional study was described in detail previously [5]. In summary, data were collected from the Nutrition, Health, and Demographic Survey of Bangladesh 2011 (NHDSBD-2011), a nationally representative survey designed to assess demographic, health, and dietary patterns across the seven administrative divisions of Bangladesh. For this study, a sub-sample of 240 women of reproductive age, 15 to 49 years, was selected from three districts (Dhaka, Khulna, and Chittagong) [18].

Socio-demographic and anthropometric data

Socio-demographic information, including age, marital status, household expenditure, food security, and lifestyle factors, was collected using a standardized, validated questionnaire. Anthropometric measurements were obtained by trained personnel following standard protocols described earlier [5]. Body mass index (BMI) was estimated using the WHO Asian cut-off [19].

Biochemical assessment

Participants fasted for at least 8 hours before venous blood collection. Samples were drawn using disposable syringes, processed to obtain serum, and transported to Dhaka on dry ice. ALT and AST activities were measured using a semi-automated biochemistry analyzer (DTN-405, DIALAB GmbH, Austria). Quality control was maintained by re-analyzing every 20th sample and repeating 12 random samples to determine the coefficient of variation (CV), calculated as CV = (SD/Mean) × 100. The CVs were 12.82% for ALT and 6.37% for AST [5].

Liver Enzyme Status

The elevated liver enzymes were defined as serum ALT > 34 U/L and AST > 31 U/L for women, consistent with established reference values [20]. Elevated liver enzymes were defined as ALT elevated or AST elevated, or both elevated [5].

Dietary data collection

Dietary data were collected by a single 24-hour recall method at the household level using a validated questionnaire [18]. Individual dietary intake was estimated by applying the Rome scale, which allocates a consumption unit (‘Man value’) to each household member based on age and sex. Males aged ≥14 years were assigned a value of 1.0; females aged ≥11 years and boys aged 11-14 years, 0.90; children aged 7-10 years, 0.75; children aged 4-6 years, 0.40; and children under 4 years, 0.15 [21,22]. Individual intake was then calculated using the following formula:

\( \text{Actual intake (g)} = \frac{\text{Family intake (g)} \times \text{Individual Man value}} {\text{Total Man value}} \)

Diet quality indicators

Food Consumption Score (FCS)

FCS was calculated for each participant using the information from the seven-day food frequency questionnaire (FFQ) according to the guidelines of the World Food Program (WFP) [23]. The dietary data were categorized into eight food groups: staples, pulses, vegetables, fruits, meat/fish, dairy, sugar, and oil (g). Each food group's consumption frequency was multiplied by standard WFP weights to obtain weighted scores, which were summed to produce the individual FCS. Participants were classified as having poor (0-28), borderline (28-42), or acceptable (>42) diets [24].

Dietary Diversity (DD)

Dietary diversity (DD) reflects the variety of foods consumed by an individual and is commonly used as an indicator of dietary intake [25]. The Minimum Dietary Diversity for Women (MDD-W) serves as a simple, dichotomous population-level indicator for assessing DD at national and subnational levels. DD was calculated using a single-day dietary assessment [26]. Foods were grouped into 10 standard categories (starchy staples, beans and peas, nuts and seeds, dairy products, flesh foods, Eggs, Dark green leafy vegetables, Vitamin A-rich fruits and vegetables, other vegetables, and other fruits). Women of reproductive age (WRA) were categorized as having inadequate DD if they consumed foods from four or fewer food groups of the 10 defined food groups in the previous 24 hours. Conversely, those who consumed at least five of the 10 food groups were considered to have adequate DD [27].

BD-Healthy Eating Index (BD-HEI)

Diet quality was collected from 24-hour recall data, which was categorized into 11 food groups using the Bangladesh Healthy Eating Index (BD-HEI), adapted from the national Food-Based Dietary Guidelines [28]. The components of the BD-HEI were categorized into adequacy, moderation, and optimum groups. Each component was scored from 0 to 5 or 10, with total BD-HEI scores ranging from 0-90. The participants with higher scores indicated better adherence to dietary recommendations. Participants were grouped into tertiles representing low, medium, and high BD-HEI categories [28].

Ethical approval

Ethical approval for this study was granted by the Institutional Review Board (IRB), Faculty of Biological Sciences, University of Dhaka (Permission No. 10/Bio.Sci./2011-2012). Written informed consent from the participants was taken before data collection.

Statistical analysis

All analyses were performed using STATA version 15.1 (Stata Corp LLC, College Station, TX, USA). Descriptive and inferential statistics were performed. Nutrient intakes, including energy and selected vitamins and minerals, were calculated using the Bangladesh Food Composition Tables [29]. Associations between liver enzyme status (normal vs. elevated) and dietary indicators (DD, FCS, BD_HEI), monthly food expenditure and place of residence were assessed using Pearson’s chi-square tests (reported as χ² = value, *p*). Fisher’s exact test was used where expected counts were <5 (reported as Fisher’s exact, *p*). Non-parametric comparisons were tested with Mann-Whitney U tests (reported as U = value, *p*). Logistic regression models were constructed to examine predictors of elevated liver enzymes while adjusting for potential confounders. Variables were included as confounders if prior inferential analyses showed *p* < 0.10. All models met the multicollinearity assumption, with variance inflation factors (VIF < 2). Statistical significance was defined as *p* < 0.05.

## Results

The study included 240 women aged 15 to 49 years. Among them, 71.3% (n = 171) had normal liver enzyme status, while 28.8% (n = 69) exhibited elevated liver enzyme status (Table 1). Socio-demographic characteristics, including age, marital status, body mass index (BMI), and food insecurity, did not differ significantly between the two groups. However, the place of residence (*p* = 0.044) and monthly food expenditure (*p* = 0.017) were significantly associated with liver enzyme status.

**Table 1:** Socio-demographic features between study participants with elevated liver enzymes (n = 244) BDT, Bangladeshi Taka; BMI, Body Mass Index: categories based on WHO Asian cut-off [19]; χ², chi-square test; *p < 0.05 considered statistically significant.

Characteristics	Liver enzyme status		Test statistic (χ²)	p-value
	Normal n (%)	Elevated n (%)		
	171 (71.3)	69 (28.8)		
Age (years)				
≤23	59(34.5)	16(23.2)	4.04	0.133
24–28	56(32.8)	31(44.9)		
≥29	56(32.8)	22(31.9)		
Marital Status				
Unmarried	10(5.8)	4(5.8)	Fisher’s exact	1.000
Married	161(94.2)	65(94.2)		
Place of residence				
Urban	38(22.2)	24(34.8)	4.05	0.044*
Rural	133(77.8)	45(65.2)		
Food Insecurity				
Never Experienced	135(78.9)	60(87.0)	2.07	0.150
Sometimes Experienced	36(21.1)	9(13.0)		
Monthly food expenditure (BDT)				
≤6,550.00	128(74.9)	40(58.0)	8.12	0.017*
6,551.00 – 9,640.00	25(14.6)	13(18.8)		
≥9,641.00	18(10.5)	16(23.2)		
Body mass index (kg/m²)				
Underweight (<18.5)	43(25.1)	10(14.7)	3.14	0.209
Normal (18.5–22.9)	84(49.1)	37(54.4)		
Overweight or obese (≥23.0)	44(25.7)	21(30.9)		

Table 2 shows differences in the prevalence and quantity of food consumption by liver enzyme status. Higher proportion of participants with elevated liver enzymes consumed milk and milk products (44.9% vs. 12.3%, *p* < 0.001), fish (79.7% vs. 66.1%, *p* = 0.037), total animal protein (91.3% vs. 79.0%, *p* = 0.023), fruits (23.2% vs. 8.8%, p = 0.003), sugar (39.1% vs. 14.0%, *p* < 0.001), and salt (26.1% vs. 14.0%, *p* = 0.026) compared to participants with normal liver enzymes. Participants with elevated liver enzymes also consumed a lower amount of rice (404.46 vs. 457.63 g/day, *p* = 0.009) and chilies (3.53 vs. 6.26 g/day, *p* = 0.001).

**Table 2:** Daily food group consumption in participants with elevated liver enzyme status IQR, interquartile range; χ², Chi-square test; Z, Mann–Whitney U test; *p < 0.05 was considered statistically significant. Food groups categorized according to the Food Composition Table for Bangladesh [29].

Food Group	Food Item	Participants consumed by a particular food group n (%)				Amount (g/day) consumed by liver enzyme status			
		Normal N = 171	Elevated N = 69	Test statistic (χ²)	p-value	Normal Median (IQR)	Elevated Median (IQR)	Test statistic (Z)	p-value
Cereals and tubers	Rice	171 (100.0)	69 (100.0)	-	N/A	457.63 (354.28 - 599.29)	404.46 (315.43 - 523.64)	2.602	0.009*
	Wheat	31 (18.1)	17 (24.6)	1.3	0.254	136.36 (17.31 - 185.93)	86.79 (39.54 - 173.08)	0.172	0.863
	Potato	121 (70.8)	48 (69.6)	0.03	0.854	90.91 (54.91 - 148.62)	109.70 (71.47 - 129.97)	−0.737	0.461
	Total	171 (100.0)	69 (100.0)	-	N/A	568.48 (448.34 - 685.96)	517.47 (395.56 - 650.35)	1.787	0.074
Vegetables	Non-Leafy Vegetables	132 (77.2)	51 (73.9)	0.29	0.589	147.25 (87.11 - 229.67)	163.64 (99.39 - 221.92)	−0.598	0.550
	Leafy Vegetables	49 (28.7)	12 (17.4)	3.29	0.070	131.71 (88.34 - 227.85)	100.00 (59.43 - 123.90)	1.851	0.064
	Other Vegetables	10 (5.9)	6 (8.7)	0.64	0.423	122.18 (65.45 - 152.54)	60.14 (19.42 - 107.46)	1.466	0.143
	Total Vegetables	146 (85.4)	56 (81.2)	0.66	0.418	187.82 (117.39 - 298.18)	177.00 (102.48 - 227.88)	0.976	0.329
Pulses	Masoor, Khesari	55 (32.2)	30 (43.5)	2.75	0.097	32.73 (16.42 - 58.44)	26.69 (13.43 - 65.45)	0.740	0.459
Milk & Milk Products	Milk and Milk Products	21 (12.3)	31 (44.9)	30.87	<0.001*	66.21 (9.82 - 123.29)	37.55 (3.91 - 110.43)	1.147	0.251
Edible Oils	Soybean Oil	161 (94.2)	68 (98.6)	2.18	0.140	17.37 (11.74 - 24.32)	15.78 (8.97 - 27.19)	1.106	0.269
	Mustard Oil	11 (6.4)	4 (5.8)	0.03	0.854	5.14 (2.43 - 15.43)	3.69 (2.12 - 9.86)	0.849	0.396
	Total	168 (98.3)	69 (100.0)	1.23	0.268	17.22 (11.74 - 24.24)	15.43 (9.15 - 27.19)	0.942	0.346
Meat, Poultry, and Eggs	Flesh food	27 (15.8)	15 (21.7)	1.21	0.272	141.82 (111.79 - 219.51)	126.00 (70.76 - 281.25)	0.368	0.713
	Eggs	32 (18.7)	14 (20.3)	0.08	0.779	32.16 (21.06 - 50.37)	32.90 (26.34 - 44.38)	−0.442	0.659
	Fish	113 (66.1)	55 (79.7)	4.35	0.037*	91.31 (51.05 - 152.54)	73.23 (38.71 - 128.75)	1.163	0.245
	Total	135 (79.0)	63 (91.3)	5.2	0.023*	113.92 (61.02 - 175.85)	108.00 (50.34 - 175.32)	0.381	0.703
Condiments and Spices	Onion	164 (95.9)	67 (97.1)	0.19	0.659	29.31 (19.37 - 42.45)	25.71 (17.31 - 44.18)	0.471	0.638
	Chili	135 (79.0)	59 (85.5)	1.37	0.243	6.26 (3.93 - 10.85)	3.53 (2.20 - 7.76)	3.377	0.001*
Fruits		15 (8.8)	16 (23.2)	9.08	0.003*	29.72 (23.48 - 114.55)	87.33 (17.38 - 190.09)	−0.474	0.635
Sugar		24 (14.0)	27 (39.1)	18.5	<0.001*	5.53 (3.45 - 9.39)	5.33 (4.14 - 10.59)	−0.444	0.657
Salt		24 (14.0)	18 (26.1)	4.95	0.026*	0.10 (0.04 - 0.28)	0.20 (0.15 - 0.28)	−1.716	0.086
Miscellaneous		33 (19.3)	20 (29.0)	2.68	0.102	28.48 (14.79 - 39.13)	33.13 (14.77 - 52.06)	−0.541	0.588

Macronutrient intake by liver enzyme status is shown in Table 3. Participants with elevated liver enzymes had a higher proportion of energy derived from total fat (*p* = 0.020), along with significantly greater intakes of saturated fatty acids (*p* = 0.010), monounsaturated fatty acids (*p* = 0.004), and dietary cholesterol (*p* = 0.015).

**Table 3:** Macronutrient intake and percentage of energy contribution by elevated liver enzymes status Z, Mann–Whitney U test; IQR, interquartile range; SFA, Saturated Fatty Acid; MUFA, Monounsaturated fatty acid; PUFA, Polyunsaturated fatty acid; *p < 0.05 was considered statistically significant. Nutrient values estimated using the Food Composition Table for Bangladesh [29].

Macronutrients	Normal Median (IQR)	Elevated Median (IQR)	Test statistic (Z)	p-value
Energy (kcal)	2157.51 (1793.24 - 2670.80)	2171.68 (1600.54 - 2583.28)	0.92	0.360
Carbohydrate (g)	410.54 (328.91 - 494.39)	397.48 (300.80 - 464.60)	1.42	0.156
% Carbohydrate	75 (70 - 78)	73 (68 - 78)	1.7	0.088
Protein (g)	62.77 (47.76 - 80.93)	58.48 (47.76 - 78.95)	0.4	0.687
% Protein	11 (9 - 13)	11 (9 - 14)	-0.82	0.415
Fats (g)	25.76 (17.86 - 37.78)	25.82 (20.97 - 41.72)	-1.14	0.256
% Fat	11 (8 - 15)	13 (9 - 17)	-2.34	0.020*
SFA (g)	4.41 (2.98 - 7.15)	5.89 (3.84 - 9.63)	-2.56	0.010*
% SFA	1.94 (1.33 - 2.79)	2.50 (1.67 - 4.36)	-3.33	0.001*
MUFA (g)	5.39 (3.66 - 8.05)	6.29 (4.50 - 9.06)	-1.98	0.047*
% MUFA	2.36 (1.66 - 3.31)	3.06 (2.01 - 4.21)	-2.91	0.004*
PUFA (g)	12.17 (7.88 - 16.37)	11.14 (7.85 - 19.58)	0.24	0.808
% PUFA	5.04 (3.65 - 6.73)	4.98 (3.56 - 7.68)	-0.4	0.687
Cholesterol (mg)	30.41 (0.00 - 106.61)	55.21 (15.21 - 137.92)	-2.44	0.015*
Dietary Fiber (g)	27.56 (20.24 - 33.98)	25.64 (18.66 - 32.93)	1.23	0.220

Table 4 shows that intakes of all assessed micronutrients did not differ significantly between participants with normal and elevated liver enzymes. Median intakes of vitamins A, D, E, C, B1, B2, B3, B6, and folate, as well as minerals including magnesium, calcium, phosphorus, sodium, potassium, zinc, and iron, were similar across groups (all *p* > 0.05).

**Table 4:** Micronutrient intake in participants with elevated liver enzyme status Z*,* Mann–Whitney U test; *p < 0.05 was considered statistically significant. Nutrient values estimated using the Food Composition Table for Bangladesh [29].

Micronutrient (Unit)	Normal (n=174) Median (IQR)	Elevated (n=70) Median (IQR)	Test statistic (Z)	P-value
Vitamin A (RAE μg)	80.53 (21.98 - 293.31)	66.30 (23.16 - 281.73)	0.24	0.812
Thiamin (B1​, mg)	4.65 (2.25 - 12.50)	3.80 (1.91 - 13.88)	0.71	0.481
Riboflavin (B2​, mg)	4.43 (3.21 - 6.60)	4.49 (3.16 - 6.75)	-0.37	0.712
Niacin (B3​, mg)	53.41 (30.31 - 94.11)	56.46 (27.30 - 83.89)	0.69	0.492
Vitamin B6​ (mg)	1.27 (0.82 - 1.64)	1.43 (0.88 - 1.73)	-0.47	0.638
Folate (B9​, μg)	0.62 (0.46 - 0.88)	0.65 (0.50 - 0.98)	-0.96	0.336
Vitamin B12​ (μg)	18.24 (10.50 - 23.81)	17.72 (9.58 - 23.63)	0.34	0.738
Vitamin C (mg)	1.56 (1.09 - 2.03)	1.60 (0.82 - 2.10)	0.61	0.545
Calcium (mg)	172.38 (123.37 - 314.91)	168.07 (116.57 - 471.05)	-0.63	0.530
Iron (mg)	348.55 (276.68 - 442.05)	333.07 (246.98 - 414.34)	0.95	0.343
Magnesium (mg)	242.88 (131.64 - 583.36)	258.06 (149.23 - 504.41)	-0.11	0.910
Zinc (mg)	1072.20 (814.59 - 1318.46)	992.05 (831.31 - 1315.48)	0.52	0.602

Table 5 shows the association of diet quality indicators with liver enzyme status. Higher proportion of participants with elevated liver enzymes consumed adequately diversified diet (≥5 food groups: 27.5% vs. 15.8%, *p* = 0.036) and poorer FCS (23.2% vs. 9.4%, *p* = 0.014), whereas no significant differences were observed in Healthy Eating Index tertiles between groups.

**Table 5:** Distribution of dietary quality indicators by liver enzyme status DD, Dietary Diversity [27]; FCS, Food Consumption Score [24]; BD_HEI, Bangladesh Healthy Eating Index [28]; *χ²,* Chi-square test; *p* < 0.05 was considered statistically significant.

Variables	Categories	Normal n (%)	Elevated n (%)	Test statistic (χ²)	P-value
DD	Inadequate (<5 food groups)	144 (84.2)	50 (72.5)	4.38	0.036*
	Adequate (≥5 or more food groups)	27 (15.8)	19 (27.5)		
FCS	Poor	16 (9.4)	16 (23.2)	8.52	0.014*
	Borderline	74 (43.3)	28 (40.6)		
	Acceptable	81 (47.4)	25 (36.2)		
BD_HEI	Tertile 1 (Low)	60 (35.1)	21 (30.4)	1.7	0.428
	Tertile 2	59 (34.5)	21 (30.4)		
	Tertile 3 (High)	52 (30.4)	27 (39.1)		

Binary logistic regression demonstrated that dietary fat intake was significantly associated with elevated liver enzymes (Table 6). In the fully adjusted model, a higher proportion of energy from fat intake was associated with increased odds of elevated liver enzymes (AOR: 1.07; 95% CI: 1.01-1.12; *p* = 0.021). Saturated fat intake (AOR: 1.05; 95% CI: 1.00-1.11; *p* = 0.038) and monounsaturated fat intake (AOR: 1.30; 95% CI: 1.08-1.57; *p* = 0.006) were also independently associated with elevated liver enzymes.

**Table 6:** Logistic regression analysis of associations between elevated liver enzyme levels and macronutrient intake COR, Crude Odds Ratio; AOR, Adjusted Odds Ratio; SFA, Saturated Fatty Acid; MUFA, Monounsaturated Fatty Acid; Model 1 adjusted for monthly food expenditure and place of residence; Model 2 adjusted for Model 1 variables and dietary diversity and Food Consumption Score; *p < 0.05 was considered statistically significant.

Variables	Crude Model		Model 1		Model 2	
	COR (95% CI)	*p*-value	AOR (95% CI)	*p*-value	AOR (95% CI)	*p*-value
% Fat	1.06 (1.01 – 1.11)	0.018	1.06 (1.00 – 1.11)	0.033	1.07 (1.01 – 1.12)	0.021
SFA (g)	1.05 (1.01 – 1.10)	0.028	1.05 (1.01 – 1.10)	0.026	1.05 (1.00 – 1.11)	0.038
% SFA	1.27 (1.09 – 1.47)	0.002	1.27 (1.09 – 1.47)	0.003	1.28 (1.09 – 1.51)	0.003
% MUFA	1.05 (0.99 – 1.11)	0.083	1.05 (0.99 – 1.12)	0.075	1.06 (1.00 – 1.13)	0.067
MUFA (g)	1.27 (1.06 – 1.51)	0.009	1.26 (1.05 – 1.50)	0.012	1.30 (1.08 – 1.57)	0.006
Cholesterol (mg)	1.00 (1.00 – 1.00)	0.028	1.00 (1.00 – 1.00)	0.027	1.00 (0.99 – 1.00)	0.085

Table 7 shows that, after adjusting for monthly food expenditure and place of residence, participants with poor FCS had more than threefold higher odds of elevated liver enzymes compared with those with acceptable FCS (AOR: 3.64; 95% CI: 1.53-8.62; *p* = 0.003). While adequate DD was associated with higher odds in the crude model (COR: 2.03; 95% CI: 1.04-3.96; *p* = 0.039), this association was no longer significant after adjustment.

**Table 7:** Logistic regression analysis of associations between FCS and elevated liver enzymes OR, Odds Ratio; AOR, Adjusted Odds Ratio; FCS, Food Consumption Score [24]; DD, Dietary Diversity [27]; *p < 0.05 was considered statistically significant.

Variables	Categories	COR	95% CI	*p*-value	AOR	95% CI	*p*-value
FCS	Acceptable	Ref			Ref		
	Borderline	1.23	0.66 – 2.29	0.523	1.39	0.72 – 2.66	0.460
	Poor	3.24	1.42 – 7.40	0.005*	3.64	1.53 – 8.62	0.003*
DD	Inadequate	Ref			Ref		
	Adequate	2.03	1.04 – 3.96	0.039*	1.72	0.83 – 3.57	0.143

## Discussion

This study investigated the association between diet quality and elevated liver enzymes among women of reproductive age in Bangladesh. Elevated liver enzymes were significantly associated with higher intake of total fat, SFA, and MUFA. Poor diet quality, indicated by low FCS, was strongly linked to elevated liver enzymes after adjusting for food expenditure, place of residence, and DD.

The present study found that higher fat intake, particularly SFA and cholesterol, was associated with a higher prevalence of elevated liver enzymes. The type and amount of fats can contribute to liver dysfunction. Saturated fatty acids (SFA) have adverse effects on lipid and glucose homeostasis, which can further aggravate liver dysfunction [30]. In Cameroonian adults, excessive fat consumption results in excess body fat accumulation, and increased visceral fat enhances the flow of free fatty acids into the liver, contributing to hepatic steatosis [31]. Among Indian adults, those with elevated liver enzymes tended to consume diets high in total fat and saturated fat but low in PUFAs [32].

In our study, participants with elevated liver enzymes consumed a higher intake of fat, especially cholesterol, reflecting poor FCS compared to those with normal liver enzymes. Similar findings were observed among Indian adults, where the intake of fat was higher among those with elevated liver enzymes as compared to those with normal liver enzymes [32]. A low FCS may be associated with an increased risk of developing non-communicable diseases (NCDs) [15].

The present study found that elevated liver enzymes were not associated with adequate DD and BD_HEI, while poor FCS was highly associated with the prevalence of elevated liver enzymes. Diet quality indices derived from single-day 24-h recall dietary intake, such as DD and BD_HEI, did not capture the actual habitual consumption of the participants; rather, 7-day FFQ-based FCS captures the day-to-day variation and usual diet [33]. To further explore the diet and liver disease association, both the 24-h recall and FFQ-based dietary assessment tools are recommended.

Overall, these findings highlight that both macronutrient composition and overall diet quality are key determinants of liver health. Interventions that promote balanced, nutrient-dense diets with adequate fruit and vegetable intake and moderate fat consumption may contribute to preventing elevated liver enzyme levels among Bangladeshi women.

Strengths and limitations

This study highlights the association between diet quality and elevated liver enzymes, using validated dietary assessment tools (24-hour recall and FFQ) and established FCS cutoffs [23]. The study has certain limitations, notably its cross-sectional design, which limits the ability to draw causal inferences. Grouping ALT-only, AST-only, and combined elevations into a single binary outcome may have masked etiologic differences among distinct liver enzyme abnormality patterns. Individual dietary intake was estimated from household-level data using the Rome scale, which, although widely applied, may result in an underestimation of intake for women due to unequal intra-household food distribution. Important confounding variables such as physical activity, alcohol intake, and viral hepatitis status could not be included due to a lack of data. In addition, the study sample was not nationally representative, which may limit the generalizability of the findings. Future research should include broader populations and longitudinal approaches.

## Conclusions

In summary, the study reveals that low FCS is associated with elevated liver enzymes among reproductive-age women in Bangladesh. High fat intake appears to adversely influence FCS, which may also be related to elevated liver enzymes among reproductive-age women in Bangladesh. Implementing targeted nutritional interventions that encourage balanced and diverse diets is essential to prevent elevated liver enzymes and related health issues.
